# 4-(2-Fluoro­phen­yl)-1-(2-oxoindolin-3-yl­idene)thio­semicarbazide

**DOI:** 10.1107/S1600536810011682

**Published:** 2010-04-02

**Authors:** Humayun Pervez, Muhammad Yaqub, Muhammad Ramzan, Mohammad S. Iqbal, M. Nawaz Tahir

**Affiliations:** a Department of Chemistry, Bahauddin Zakariya University, Multan 60800, Pakistan; b Department of Chemistry, Government College University, Lahore, Pakistan; c Department of Physics, University of Sargodha, Sargodha, Pakistan

## Abstract

The title compound, C_15_H_11_FN_4_OS, is almost planar, the dihedral angle between the aromatic ring systems being 5.00 (13)°. The conformation is stabilized by intra­molecular N—H⋯N and N—H⋯O hydrogen bonds, which generate *S*(5) and *S*(6) rings, respectively. N—H⋯F and C—H⋯S inter­actions also occur. In the crystal, inversion dimers linked by pairs of N—H⋯O hydrogen bonds occur, generating *R*_2_^2^(8) loops.

## Related literature

For related structures and medicinal background, see: Pervez *et al.* (2009 ▶, 2010 ▶). For graph-set theory, see: Bernstein *et al.* (1995 ▶).
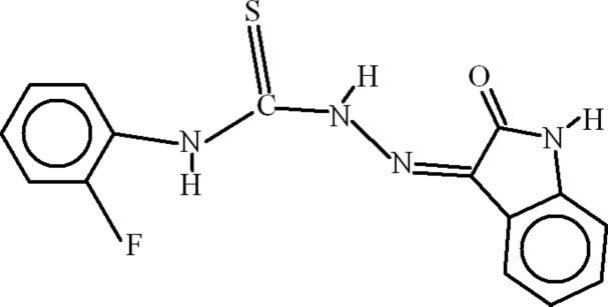


## Experimental

### 

#### Crystal data

C_15_H_11_FN_4_OS
*M* *_r_* = 314.34
Monoclinic, 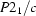

*a* = 5.7646 (3) Å
*b* = 18.4939 (12) Å
*c* = 13.6772 (8) Å
β = 91.212 (3)°
*V* = 1457.80 (15) Å^3^
*Z* = 4
Mo *K*α radiation
μ = 0.24 mm^−1^
*T* = 296 K
0.30 × 0.14 × 0.12 mm

#### Data collection

Bruker Kappa APEXII CCD diffractometer
Absorption correction: multi-scan (*SADABS*; Bruker, 2005 ▶) *T*_min_ = 0.963, *T*_max_ = 0.971
11407 measured reflections
2626 independent reflections
1437 reflections with *I* > 2σ(*I*)
*R*_int_ = 0.068

#### Refinement

*R*[*F* ^2^ > 2σ(*F* ^2^)] = 0.047
*wR*(*F* ^2^) = 0.114
*S* = 0.96
2626 reflections
199 parameters
H-atom parameters constrained
Δρ_max_ = 0.15 e Å^−3^
Δρ_min_ = −0.22 e Å^−3^

### 

Data collection: *APEX2* (Bruker, 2007 ▶); cell refinement: *SAINT* (Bruker, 2007 ▶); data reduction: *SAINT*; program(s) used to solve structure: *SHELXS97* (Sheldrick, 2008 ▶); program(s) used to refine structure: *SHELXL97* (Sheldrick, 2008 ▶); molecular graphics: *ORTEP-3 for Windows* (Farrugia, 1997 ▶) and *PLATON* (Spek, 2009 ▶); software used to prepare material for publication: *WinGX* (Farrugia, 1999 ▶) and *PLATON*.

## Supplementary Material

Crystal structure: contains datablocks global, I. DOI: 10.1107/S1600536810011682/hb5381sup1.cif

Structure factors: contains datablocks I. DOI: 10.1107/S1600536810011682/hb5381Isup2.hkl

Additional supplementary materials:  crystallographic information; 3D view; checkCIF report

## Figures and Tables

**Table 1:** Hydrogen-bond geometry (Å, °)

*D*—H⋯*A*	*D*—H	H⋯*A*	*D*⋯*A*	*D*—H⋯*A*
N1—H1⋯O1^i^	0.86	2.07	2.912 (3)	164
N3—H3⋯O1	0.86	2.08	2.762 (3)	135
N4—H4*A*⋯F1	0.86	2.21	2.613 (2)	109
N4—H4*A*⋯N2	0.86	2.13	2.585 (3)	113
C15—H15⋯S1	0.93	2.56	3.216 (3)	128

Symmetry code: (i) 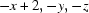.

**Fig. 1.**
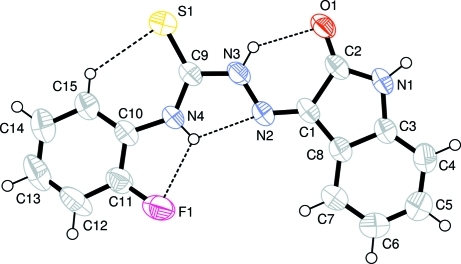
View of (I) with ellipsoids drawn at the 50% probability level. The dotted lines indicate the intramolecular H-bonds.

**Fig. 2.**
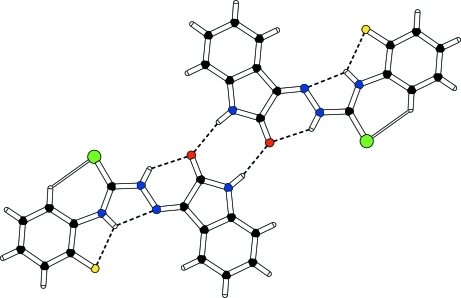
The partial packing of (I), which shows that molecules form inversion dimers.

## supplementary crystallographic information

### Comment

As part of our ongoing studies of N^4^-arylsubstituted isatins-3-thiosemecarbazones with certain medicinal applications (Pervez *et al.*, 2009, 2010), we now report the synthesis and crystal structure of the title compound (I, Fig. 1).

The crystal structure of (II) i.e. 1-(5-nitro-2-oxoindolino-3-ylidene)- 4-*o*-tolylthiosemicarbazide methanol monosolvate (Pervez *et al.*, 2009) has been published. The title compound (I) differs from (II) due to the absence of nitro function at position-5 of the isatin scaffold and presence of fluoro instead of methyl group at position-2 of the phenyl ring substituted at N^4^ of the thiosemicarbazone moiety. In (I), the 2-oxoindolin A (C1–C8/N1/O1), thiosemicarbazide B (N2/N3/C9/S1/N4) and the 2-fluorophenyl C (C10—C15/F1) are planar with maximum r. m. s. deviations of 0.0289, 0.0261 and 0.0056 Å, respectively. Due to intramolecular H-bondings (Table 1, Fig. 1), two S(5) and two S(6) (Bernstein *et al.*, 1995) ring motifs are formed. The molecules are dimerised due to intermolecular H-bonding of N—H···O type with R_2_^2^(8) ring motifs.

### Experimental

To a hot solution of isatin (0.74 g, 5.0 mmol) in ethanol (10 ml) containing a few drops of glacial acetic acid was added 4-*o* -fluorophenylthiosemicarbazide (0.93 g, 5.0 mmol) dissolved in ethanol (10 ml) under stirring. The reaction mixture was then heated under reflux for 2 h. The yellow crystalline solid formed during refluxing was collected by suction filtration. Thorough washing with hot ethanol followed by ether furnished the target compound (I) in pure form (1.20 g, 76 %), m.p. 505-507 K (d). The dark yellow needles of (I) were grown in ethyl acetate-petroleum ether (1:5) system by diffusion method at room temperature.

### Crystal data

**Table:**

C_15_H_11_FN_4_OS	*F*(000) = 648
*M**_r_* = 314.34	*D*_x_ = 1.432 Mg m^−^^3^
Monoclinic, *P*2_1_/*c*	Mo *K*α radiation, λ = 0.71073 Å
Hall symbol: -P 2ybc	Cell parameters from 2626 reflections
*a* = 5.7646 (3) Å	θ = 3.5–25.3°
*b* = 18.4939 (12) Å	µ = 0.24 mm^−^^1^
*c* = 13.6772 (8) Å	*T* = 296 K
β = 91.212 (3)°	Needle, dark yellow
*V* = 1457.80 (15) Å^3^	0.30 × 0.14 × 0.12 mm
*Z* = 4	

### Data collection

**Table:**

Bruker Kappa APEXII CCD diffractometer	2626 independent reflections
Radiation source: fine-focus sealed tube	1437 reflections with *I* > 2σ(*I*)
graphite	*R*_int_ = 0.068
Detector resolution: 7.80 pixels mm^-1^	θ_max_ = 25.3°, θ_min_ = 3.5°
ω scans	*h* = −6→6
Absorption correction: multi-scan (*SADABS*; Bruker, 2005)	*k* = −21→22
*T*_min_ = 0.963, *T*_max_ = 0.971	*l* = −16→16
11407 measured reflections	

### Refinement

**Table:**

Refinement on *F*^2^	Primary atom site location: structure-invariant direct methods
Least-squares matrix: full	Secondary atom site location: difference Fourier map
*R*[*F*^2^ > 2σ(*F*^2^)] = 0.047	Hydrogen site location: inferred from neighbouring sites
*wR*(*F*^2^) = 0.114	H-atom parameters constrained
*S* = 0.96	*w* = 1/[σ^2^(*F*_o_^2^) + (0.0467*P*)^2^] where *P* = (*F*_o_^2^ + 2*F*_c_^2^)/3
2626 reflections	(Δ/σ)_max_ < 0.001
199 parameters	Δρ_max_ = 0.15 e Å^−^^3^
0 restraints	Δρ_min_ = −0.22 e Å^−^^3^

### Special details

**Table:**

Geometry. Bond distances, angles etc. have been calculated using the rounded fractional coordinates. All su's are estimated from the variances of the (full) variance-covariance matrix. The cell esds are taken into account in the estimation of distances, angles and torsion angles
Refinement. Refinement of *F*^2^ against ALL reflections. The weighted *R*-factor *wR* and goodness of fit *S* are based on *F*^2^, conventional *R*-factors *R* are based on *F*, with *F* set to zero for negative *F*^2^. The threshold expression of *F*^2^ > σ(*F*^2^) is used only for calculating *R*-factors(gt) *etc*. and is not relevant to the choice of reflections for refinement. *R*-factors based on *F*^2^ are statistically about twice as large as those based on *F*, and *R*- factors based on ALL data will be even larger.

### Fractional atomic coordinates and isotropic or equivalent isotropic displacement parameters (Å^2^)

**Table:**

	*x*	*y*	*z*	*U*_iso_*/*U*_eq_	
S1	0.09191 (15)	0.20047 (5)	0.00689 (6)	0.0742 (3)	
F1	−0.0601 (3)	0.14317 (12)	0.37578 (11)	0.0911 (9)	
O1	0.7157 (3)	0.04818 (11)	0.01234 (13)	0.0575 (8)	
N1	0.9081 (4)	−0.02584 (13)	0.12316 (16)	0.0547 (9)	
N2	0.4000 (4)	0.06836 (12)	0.18384 (15)	0.0489 (8)	
N3	0.3340 (4)	0.10541 (12)	0.10344 (15)	0.0510 (8)	
N4	0.0393 (3)	0.15113 (12)	0.19057 (14)	0.0499 (8)	
C1	0.5848 (5)	0.02916 (15)	0.17922 (19)	0.0474 (10)	
C2	0.7385 (5)	0.01971 (16)	0.0932 (2)	0.0494 (11)	
C3	0.8851 (5)	−0.04562 (15)	0.2217 (2)	0.0513 (10)	
C4	1.0273 (5)	−0.08819 (17)	0.2785 (2)	0.0696 (12)	
C5	0.9689 (6)	−0.09758 (19)	0.3747 (3)	0.0803 (14)	
C6	0.7757 (6)	−0.06518 (19)	0.4128 (2)	0.0774 (15)	
C7	0.6305 (5)	−0.02238 (17)	0.3550 (2)	0.0658 (11)	
C8	0.6871 (4)	−0.01264 (15)	0.25825 (19)	0.0494 (10)	
C9	0.1480 (5)	0.15218 (15)	0.10471 (18)	0.0486 (10)	
C10	−0.1520 (4)	0.19126 (15)	0.22268 (19)	0.0479 (10)	
C11	−0.2029 (5)	0.18597 (18)	0.3198 (2)	0.0626 (11)	
C12	−0.3861 (6)	0.2210 (2)	0.3614 (3)	0.0864 (14)	
C13	−0.5248 (6)	0.2633 (2)	0.3026 (3)	0.0849 (16)	
C14	−0.4818 (5)	0.26920 (18)	0.2062 (3)	0.0743 (14)	
C15	−0.2977 (5)	0.23337 (16)	0.1658 (2)	0.0623 (11)	
H1	1.01726	−0.04097	0.08643	0.0657*	
H3	0.40914	0.09984	0.05030	0.0611*	
H4	1.15840	−0.10996	0.25313	0.0834*	
H4A	0.09573	0.12107	0.23275	0.0598*	
H5	1.06218	−0.12647	0.41499	0.0960*	
H6	0.74193	−0.07212	0.47828	0.0929*	
H7	0.49911	−0.00091	0.38065	0.0791*	
H12	−0.41496	0.21619	0.42776	0.1037*	
H13	−0.64912	0.28809	0.32902	0.1015*	
H14	−0.57770	0.29779	0.16668	0.0892*	
H15	−0.27161	0.23771	0.09919	0.0747*	

### Atomic displacement parameters (Å^2^)

**Table:**

	*U*^11^	*U*^22^	*U*^33^	*U*^12^	*U*^13^	*U*^23^
S1	0.0779 (6)	0.0878 (7)	0.0577 (5)	0.0167 (5)	0.0193 (4)	0.0148 (5)
F1	0.0833 (14)	0.1408 (19)	0.0495 (10)	0.0171 (13)	0.0103 (9)	−0.0024 (11)
O1	0.0453 (12)	0.0735 (15)	0.0543 (12)	0.0021 (10)	0.0167 (9)	−0.0014 (11)
N1	0.0398 (14)	0.0640 (17)	0.0610 (15)	0.0076 (12)	0.0148 (11)	−0.0042 (13)
N2	0.0410 (14)	0.0541 (15)	0.0519 (14)	0.0019 (12)	0.0100 (11)	−0.0051 (12)
N3	0.0481 (14)	0.0577 (16)	0.0477 (14)	0.0049 (12)	0.0153 (11)	−0.0039 (12)
N4	0.0460 (14)	0.0585 (16)	0.0455 (13)	0.0098 (12)	0.0118 (11)	−0.0016 (11)
C1	0.0397 (16)	0.0486 (18)	0.0544 (17)	−0.0033 (14)	0.0127 (13)	−0.0092 (14)
C2	0.0399 (17)	0.055 (2)	0.0537 (18)	−0.0072 (15)	0.0124 (13)	−0.0104 (15)
C3	0.0417 (17)	0.0550 (19)	0.0574 (18)	0.0009 (15)	0.0072 (14)	−0.0036 (16)
C4	0.054 (2)	0.076 (2)	0.079 (2)	0.0149 (18)	0.0073 (17)	0.0046 (19)
C5	0.075 (2)	0.089 (3)	0.077 (2)	0.017 (2)	0.0008 (19)	0.012 (2)
C6	0.086 (3)	0.090 (3)	0.0564 (19)	0.010 (2)	0.0068 (18)	0.0090 (19)
C7	0.065 (2)	0.077 (2)	0.0558 (19)	0.0075 (19)	0.0142 (16)	−0.0024 (17)
C8	0.0425 (17)	0.0527 (19)	0.0532 (17)	0.0001 (14)	0.0055 (13)	−0.0062 (15)
C9	0.0448 (17)	0.0512 (18)	0.0504 (16)	−0.0002 (15)	0.0124 (13)	−0.0052 (14)
C10	0.0404 (16)	0.0517 (19)	0.0518 (17)	−0.0038 (15)	0.0073 (13)	−0.0137 (14)
C11	0.0508 (19)	0.085 (2)	0.0519 (18)	0.0027 (18)	0.0005 (15)	−0.0134 (17)
C12	0.065 (2)	0.131 (3)	0.064 (2)	0.001 (2)	0.0214 (18)	−0.036 (2)
C13	0.049 (2)	0.106 (3)	0.100 (3)	0.008 (2)	0.010 (2)	−0.044 (2)
C14	0.053 (2)	0.075 (2)	0.095 (3)	0.0098 (18)	0.0041 (18)	−0.016 (2)
C15	0.0504 (18)	0.069 (2)	0.068 (2)	0.0058 (17)	0.0099 (15)	−0.0038 (17)

### Geometric parameters (Å, °)

**Table:**

S1—C9	1.635 (3)	C5—C6	1.377 (5)
F1—C11	1.365 (4)	C6—C7	1.387 (4)
O1—C2	1.229 (3)	C7—C8	1.381 (4)
N1—C2	1.348 (4)	C10—C15	1.375 (4)
N1—C3	1.406 (4)	C10—C11	1.370 (4)
N2—N3	1.344 (3)	C11—C12	1.373 (5)
N2—C1	1.291 (4)	C12—C13	1.368 (5)
N3—C9	1.378 (4)	C13—C14	1.351 (6)
N4—C9	1.343 (3)	C14—C15	1.377 (4)
N4—C10	1.407 (3)	C4—H4	0.9300
N1—H1	0.8600	C5—H5	0.9300
N3—H3	0.8600	C6—H6	0.9300
N4—H4A	0.8600	C7—H7	0.9300
C1—C8	1.445 (4)	C12—H12	0.9300
C1—C2	1.498 (4)	C13—H13	0.9300
C3—C8	1.396 (4)	C14—H14	0.9300
C3—C4	1.367 (4)	C15—H15	0.9300
C4—C5	1.376 (5)		
			
S1···C15	3.216 (3)	C9···C2^vi^	3.403 (4)
S1···C13^i^	3.661 (4)	C9···C14^iii^	3.323 (4)
S1···C11^ii^	3.696 (3)	C9···O1^vi^	3.372 (3)
S1···C12^ii^	3.665 (4)	C10···C1^vi^	3.407 (4)
S1···H15	2.5600	C11···S1^vii^	3.696 (3)
S1···H13^i^	2.8900	C12···S1^vii^	3.665 (4)
F1···N4	2.613 (2)	C13···S1^viii^	3.661 (4)
F1···H4A	2.2100	C14···C9^vi^	3.323 (4)
O1···N2	3.022 (3)	C15···N3^vi^	3.280 (4)
O1···N3	2.762 (3)	C15···S1	3.216 (3)
O1···C9^iii^	3.372 (3)	C2···H3	2.4700
O1···N3^iv^	3.262 (3)	C2···H3^iv^	3.0600
O1···O1^iv^	3.072 (3)	C2···H1^v^	2.8800
O1···N1^v^	2.912 (3)	C5···H14^ix^	3.0200
O1···C2^iv^	3.218 (3)	C6···H14^ix^	2.9800
O1···H3	2.0800	C9···H15	2.8900
O1···H1^v^	2.0700	C14···H4^x^	2.9600
N1···O1^v^	2.912 (3)	H1···O1^v^	2.0700
N2···O1	3.022 (3)	H1···C2^v^	2.8800
N2···N4	2.585 (3)	H3···O1	2.0800
N3···O1^iv^	3.262 (3)	H3···C2	2.4700
N3···O1	2.762 (3)	H3···C2^iv^	3.0600
N3···C15^iii^	3.280 (4)	H4···C14^xi^	2.9600
N4···C2^vi^	3.254 (4)	H4A···F1	2.2100
N4···F1	2.613 (2)	H4A···N2	2.1300
N4···N2	2.585 (3)	H13···S1^viii^	2.8900
N2···H4A	2.1300	H14···C5^xii^	3.0200
C1···C10^iii^	3.407 (4)	H14···C6^xii^	2.9800
C2···N4^iii^	3.254 (4)	H15···S1	2.5600
C2···C9^iii^	3.403 (4)	H15···C9	2.8900
C2···O1^iv^	3.218 (3)		
			
C2—N1—C3	111.8 (2)	S1—C9—N4	129.5 (2)
N3—N2—C1	117.8 (2)	C11—C10—C15	116.6 (2)
N2—N3—C9	121.1 (2)	N4—C10—C15	126.6 (2)
C9—N4—C10	130.4 (2)	N4—C10—C11	116.8 (2)
C3—N1—H1	124.00	C10—C11—C12	123.4 (3)
C2—N1—H1	124.00	F1—C11—C10	116.5 (2)
N2—N3—H3	119.00	F1—C11—C12	120.1 (3)
C9—N3—H3	119.00	C11—C12—C13	118.1 (4)
C10—N4—H4A	115.00	C12—C13—C14	120.2 (3)
C9—N4—H4A	115.00	C13—C14—C15	120.8 (3)
N2—C1—C2	127.3 (2)	C10—C15—C14	120.8 (3)
C2—C1—C8	106.6 (2)	C3—C4—H4	121.00
N2—C1—C8	126.0 (2)	C5—C4—H4	121.00
N1—C2—C1	105.8 (2)	C4—C5—H5	119.00
O1—C2—N1	127.2 (3)	C6—C5—H5	119.00
O1—C2—C1	127.1 (3)	C5—C6—H6	119.00
N1—C3—C4	128.9 (3)	C7—C6—H6	119.00
N1—C3—C8	108.9 (2)	C6—C7—H7	121.00
C4—C3—C8	122.2 (3)	C8—C7—H7	121.00
C3—C4—C5	117.4 (3)	C11—C12—H12	121.00
C4—C5—C6	121.6 (3)	C13—C12—H12	121.00
C5—C6—C7	121.1 (3)	C12—C13—H13	120.00
C6—C7—C8	117.9 (3)	C14—C13—H13	120.00
C1—C8—C3	106.9 (2)	C13—C14—H14	120.00
C3—C8—C7	119.8 (2)	C15—C14—H14	120.00
C1—C8—C7	133.3 (2)	C10—C15—H15	120.00
S1—C9—N3	118.23 (19)	C14—C15—H15	120.00
N3—C9—N4	112.3 (2)		
			
C3—N1—C2—O1	177.5 (3)	C8—C3—C4—C5	−0.2 (5)
C3—N1—C2—C1	−1.8 (3)	N1—C3—C8—C1	−0.2 (3)
C2—N1—C3—C4	−177.2 (3)	N1—C3—C8—C7	−178.3 (3)
C2—N1—C3—C8	1.3 (3)	C4—C3—C8—C1	178.5 (3)
C1—N2—N3—C9	−175.5 (3)	C4—C3—C8—C7	0.3 (4)
N3—N2—C1—C2	0.0 (4)	C3—C4—C5—C6	−0.4 (5)
N3—N2—C1—C8	178.0 (2)	C4—C5—C6—C7	0.8 (5)
N2—N3—C9—S1	174.9 (2)	C5—C6—C7—C8	−0.7 (5)
N2—N3—C9—N4	−4.4 (4)	C6—C7—C8—C1	−177.4 (3)
C10—N4—C9—S1	−0.8 (4)	C6—C7—C8—C3	0.1 (4)
C10—N4—C9—N3	178.3 (2)	N4—C10—C11—F1	1.1 (4)
C9—N4—C10—C11	−170.6 (3)	N4—C10—C11—C12	−179.0 (3)
C9—N4—C10—C15	11.8 (4)	C15—C10—C11—F1	179.0 (3)
N2—C1—C2—O1	0.6 (5)	C15—C10—C11—C12	−1.1 (5)
N2—C1—C2—N1	180.0 (3)	N4—C10—C15—C14	179.0 (3)
C8—C1—C2—O1	−177.7 (3)	C11—C10—C15—C14	1.3 (4)
C8—C1—C2—N1	1.7 (3)	F1—C11—C12—C13	−180.0 (3)
N2—C1—C8—C3	−179.2 (3)	C10—C11—C12—C13	0.1 (5)
N2—C1—C8—C7	−1.5 (5)	C11—C12—C13—C14	0.7 (5)
C2—C1—C8—C3	−0.9 (3)	C12—C13—C14—C15	−0.5 (5)
C2—C1—C8—C7	176.9 (3)	C13—C14—C15—C10	−0.6 (5)
N1—C3—C4—C5	178.1 (3)		

Symmetry codes: (i) *x*+1, −*y*+1/2, *z*−1/2; (ii) *x*, −*y*+1/2, *z*−1/2; (iii) *x*+1, *y*, *z*; (iv) −*x*+1, −*y*, −*z*; (v) −*x*+2, −*y*, −*z*; (vi) *x*−1, *y*, *z*; (vii) *x*, −*y*+1/2, *z*+1/2; (viii) *x*−1, −*y*+1/2, *z*+1/2; (ix) −*x*, *y*−1/2, −*z*+1/2; (x) −*x*+1, *y*+1/2, −*z*+1/2; (xi) −*x*+1, *y*−1/2, −*z*+1/2; (xii) −*x*, *y*+1/2, −*z*+1/2.

### Hydrogen-bond geometry (Å, °)

**Table:**

*D*—H···*A*	*D*—H	H···*A*	*D*···*A*	*D*—H···*A*
N1—H1···O1^v^	0.86	2.07	2.912 (3)	164
N3—H3···O1	0.86	2.08	2.762 (3)	135
N4—H4A···F1	0.86	2.21	2.613 (2)	109
N4—H4A···N2	0.86	2.13	2.585 (3)	113
C15—H15···S1	0.93	2.56	3.216 (3)	128

Symmetry codes: (v) −*x*+2, −*y*, −*z*.
